# Update of Modified Version of the Foot Function Index Tool Spanish Version (FFI-Sp), in Patients with Rheumatoid Arthritis: Cross Sectional Study

**DOI:** 10.3390/medicina60081339

**Published:** 2024-08-18

**Authors:** Maria Gamez-Guijarro, Andres Reinoso-Cobo, Luis M. Gordillo-Fernandez, Mercedes Ortiz-Romero, Ana Belen Ortega-Avila, Esther Chicharro-Luna, Gabriel Gijon-Nogueron, Eva Lopezosa-Reca

**Affiliations:** 1 Department of Nursing and Podiatry, Faculty of Health Sciences, University of Malaga, Arquitecto Francisco Peñalosa 3, Ampliación de Campus de Teatinos, 29071 Malaga, Spain; mgamez303@uma.es (M.G.-G.); anaortavi@uma.es (A.B.O.-A.); gagijon@uma.es (G.G.-N.); evalopezosa@uma.es (E.L.-R.); 2 Department of Podiatry, Universidad de Sevilla, 41009 Sevilla, Spain; lgordillo@us.es (L.M.G.-F.); mortiz17@us.es (M.O.-R.); 3 IBIMA Plataforma BIONAND, 29010 Malaga, Spain; 4 Department Behavioral Sciences and Health, Nursing Area, Faculty of Medicine, University Miguel Hernández, 03202 Alicante, Spain; ec.luna@umh.es

**Keywords:** foot, function, rheumatoid arthritis, patient-reported outcome measure

## Abstract

*Background and Objectives*: The Foot Function Index (FFI) is a widely recognized patient-reported outcome measure (PROM) for assessing foot functionality and its impact on quality of life in individuals with rheumatoid arthritis (RA). This study aimed to observe the behavior of the tool in the Spanish population with RA, optimize the tool, and check its functionality. *Materials and Methods*: A total of 549 RA patients, with a predominant female participation (75.6%). This study involved a comprehensive statistical analysis, leading to a refined version of the FFI for a Spanish-speaking population. *Results*: The original 23-item FFI was revised, resulting in a 15-item version by excluding items that caused confusion or were considered redundant. This modified version maintained the original’s subscales of pain, disability, and activity limitation, but with an adjusted item distribution. The construct validity was confirmed through exploratory factor analysis, demonstrating excellent fit indices (Kaiser–Meyer–Olkin test = 0.926, Bartlett’s test of sphericity = 4123.48, *p* < 0.001). The revised FFI demonstrated good internal consistency (Cronbach’s alpha = 0.96) and test–retest reliability (ICC = 0.89). *Conclusions*: This study highlights the applicability of the FFI in Spanish-speaking RA populations, offering a valid and reliable tool for clinicians and researchers. The modifications enhance the FFI’s relevance for RA patients, facilitating better assessment and management of foot-related functional impairments.

## 1. Introduction

Rheumatoid arthritis (RA) is delineated as a chronic and systemic autoimmune malady, characterized predominantly by its inflammatory ramifications on synovial articulations alongside various extra-articular manifestations. This ailment principally afflicts synovial joints, commencing its onslaught on the smaller peripheral joints before potentially progressing to the larger proximal joints in the absence of medical intervention [1]. The disease is characterized by persistent symmetric polyarthritis (synovitis) that affects the hands and feet, although any joint lined by a synovial membrane may be involved [2].

The precise pathogenesis of RA continues to be a subject of ongoing investigation; however, it is postulated to emanate from an intricate interplay between genetic predispositions and environmental precipitants, notably smoking [3]. There is currently no cure for RA, and the treatment goals are to reduce pain and stop/slow further joint damage. Treatment options may include medications, physical therapy, and lifestyle changes such as maintaining a healthy weight and participating in regular exercise [4]. Lab tests such as erythrocyte sedimentation rate (ESR), C-reactive protein (CRP), rheumatoid factor (RF), and antibodies to cyclic citrullinated peptides (CCPs) can help confirm the presence of inflammation and aid in the diagnosis [5].

Patients with rheumatoid arthritis (RA) may experience a loss of functionality due to joint inflammation and pain, which can lead to difficulty performing daily activities such as dressing, grooming, and cooking, which can further impact a patient’s overall functionality. It is important for healthcare professionals to assess a patient’s functional status and provide appropriate interventions to improve their quality of life [6].

RA can have a significant impact on foot function and can affect the mobility of these patients, causing pain, soreness, and difficulty performing daily activities such as walking and standing. In addition, RA can also lead to deformities in the feet, such as hallux valgus and claw toes, which can further impact foot function [7,8].

Assessing foot function in RA patients is crucial for effective treatment and management of the disease. Foot function assessment can be performed using tools such as the Foot Function Index-Revised Short Form (FFI-RS) questionnaire, which can help healthcare professionals identify foot problems and provide appropriate interventions [9].

The Foot Function Index (FFI) is a widely used tool for assessing disability in patients with various foot conditions. Its translation and validation into Spanish enable its use within the Spanish-speaking community, allowing for the comparison of results across different studies and patient groups [10].

Therefore, our objective is to observe the behavior of the tool in the Spanish population with AR, optimize the tool, and check its functionality.

## 2. Materials and Methods

### 2.1. Design

Observational Study.

### 2.2. Participants

A total of 549 patients with foot pain and rheumatoid arthritis were included, following the classification criteria approved by the American College of Rheumatology and the European League Against Rheumatism in 2010 [11]. These patients were diagnosed by the rheumatology service of Hospital Virgen de las Nieves. The enrollment period for these patients at hospital outpatient clinics was from January 2018 to April 2023. The inclusion criteria specified adults (more than 18 years old) with foot pain greater than 3 on a Visual Analogue Scale (VAS) for foot pain and rheumatoid arthritis (RA) (supplementary file S1).

Exclusion criteria included the presence of other musculoskeletal diseases (such as lupus, scleroderma, psoriasis arthritis…), central or peripheral nervous system disorders, or endocrine disorders such as diabetes mellitus.

### 2.3. Procedure

Patients who fulfilled the inclusion criteria were approached by members of the rheumatology service at Hospital Virgen de las Nieves (Granada, Spain) during their regular visits and informed about the ongoing study and their potential participation. All those who agreed were provided with an information sheet and invited to take part. Participants who consented were interviewed and given additional study details. Written consent was obtained from all participants before the interviews began.

### 2.4. Data Collection

#### Demographic and Clinical Characteristics

Demographic characteristics recorded included patient age, gender, disease duration, and current therapy. Clinical data were obtained using the Spanish version of the Foot Function Index (FFI-Sp)(Annex 1 with 23 items [12]), a questionnaire currently consisting of 23 questions with a Likert scale ranging from 0 to 10 which is used to evaluate three domains: functionality, pain, and physical activity. In this adapted version for patients with rheumatoid arthritis, the total score ranges from 0 to 100, where a score of 0 indicates positive outcomes and a score closer to 100 represents more negative outcomes. In this adapted version, it is intended for use with patients with rheumatoid arthritis together with the visual analogue scale for pain (VAS pain), which is both general and specific to the foot [13]. These scales will be used as a control measure and for correlation with the FFI-Sp.

### 2.5. Statistical Analysis

All statistical analyses were performed using SPSS v.28.0 (IBM, International Business Machines Corporation, Armonk, NY, USA) and JAMOVI statistical software version 2.3 (2022). The Kolmogorov–Smirnov test was employed to assess the normality of distributions, and mean values and standard deviations were calculated for descriptive statistics.

Construct validity was evaluated through exploratory factor analysis in every case, and a confirmatory factor analysis (CFA) was conducted to assess the factor structure. The model fit was assessed using several criteria: the chi-square/df function, where a value of less than 3 indicated a good fit; the root mean square error of approximation (RMSEA) with 90% confidence intervals, where values between 0.05 and 0.07 were acceptable; the comparative fit index (CFI); the goodness of fit index (GFI); and the normed fit index (NFI). For these indices, a value of 0.90 was considered indicative of good fit. Multi-normality was evaluated using Mardia’s coefficient (multivariate kurtosis), which should not exceed “p” (p + 2), where “p” represents the number of observed variables.

Test–retest reliability was assessed using intraclass correlation coefficients (ICC 2,1). The Spanish version of the FFI was administered twice by the same observer, with a 7-day interval between tests. An ICC 2,1 value greater than 0.7 was classified as “excellent”, 0.60–0.74 as “good”, 0.40–0.59 as “fair”, and less than 0.40 as “poor” [14]. The absolute reliability of the Spanish version of the FFI was evaluated using the standard error of measurement (SEM) and the minimal detectable change (MDC) [15,16].

Internal consistency was measured using Cronbach’s alpha. Values greater than 0.7 indicated ‘fair’ internal consistency, those between 0.8 and 0.9 were rated as ‘good’, and values above 0.9 were considered ‘excellent’ [17].

Convergent validity between the Spanish version of the FFI questionnaire and the VAS pain scales (general and foot-specific) was assessed using Pearson’s correlations. Coefficients less than 0.30 indicated poor correlations, those below 0.60 indicated moderate correlations, and coefficients greater than 0.60 indicated strong correlations [18]. Furthermore, the Flesch Reading Ease test and the Flesch–Kincaid Grade Level test were conducted to determine the readability of the text [19].

The FFI-Sp was deemed to exhibit floor and ceiling effects if more than 15% of participants scored the minimum and maximum possible scores, respectively, on the questionnaire [17].

## 3. Results

This study encompassed a cohort of 549 participants, of which 75.6% (*n* = 415) were female and 24.4% (*n* = 134) were male. The participants had an average disease duration of 15.93 years (SD = 11.06) and a mean age of 59.6 years (SD = 12.70). Table 1 delineates the demographic and clinical characteristics of the sample population in detail.

The readability of the text was evaluated using the Flesch Reading Ease test and the Flesch–Kincaid Grade Level test. The Flesch Reading Ease score was 33.1, indicating that the text is fairly easy to read. The Flesch–Kincaid Grade Level was 20.3, suggesting the text is suitable for readers at a 7th- to 8th-grade reading level.

### 3.1. Construct Validity

In our exploratory factor analysis, the results from the Kaiser–Meyer–Olkin test (0.926) and Bartlett’s test of sphericity (4123.48) (*p* < 0.001) indicated that the correlation matrix for the validity of FFI-Sp-RA (Annex 2) was appropriate. (Table 2).

**Figure 1:**
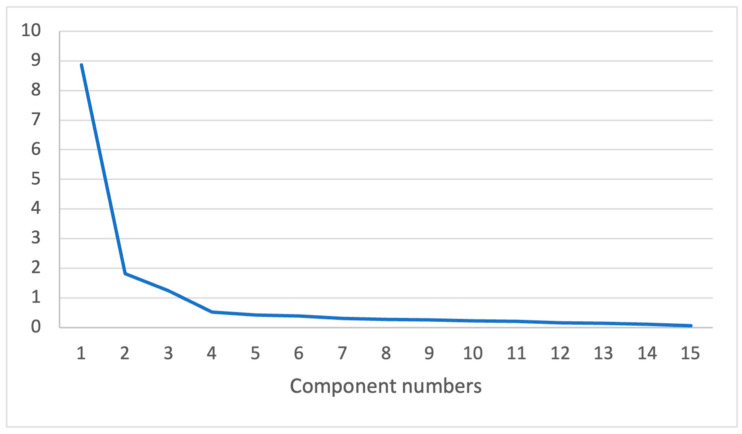
Screen plot.

This analysis yielded a three-factor solution, as illustrated in the scree plot (Figure 1), which explained 79.05% of the total variance.

The questionnaire items were categorized into three factors: Factor 1, information about foot pain (items 1, 2, 3, 4, 7, 8, 9); Factor 2, information on the impact of disability (items 11, 13, 14, 16, 17, 18); and Factor 3, information on physical activity (items 22, 23). The confirmatory factor analysis (CFA) yielded the following results: relative chi-square (χ^2^/df; 52.72, *p* < 0.001), RMSEA 0.0966, CFI 0.943, GFI 0.931, and NFI 0.94. (Table 3).

**Figure 2:**
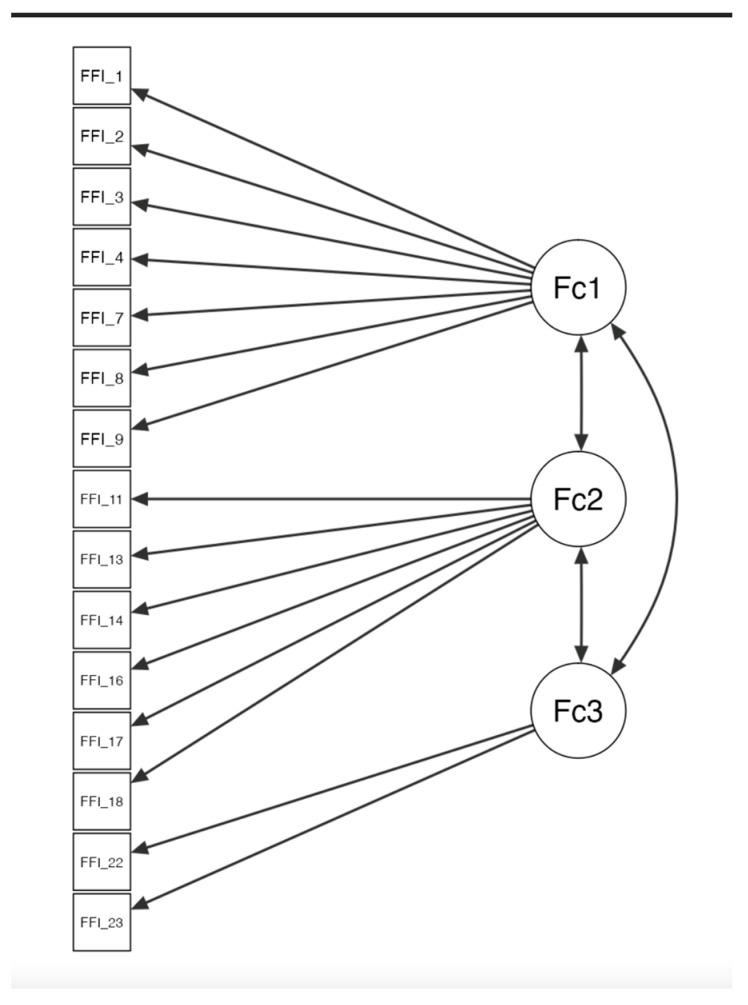
Confirmatory factor structure of FFI-Sp-RA. Fc1: Factor 1; Fc2: Factor 2; Fc3: Factor 3.

These findings indicate that the model exhibits excellent goodness of fit and meets the criteria for multi-normality (Figure 2).

**Figure 3:**
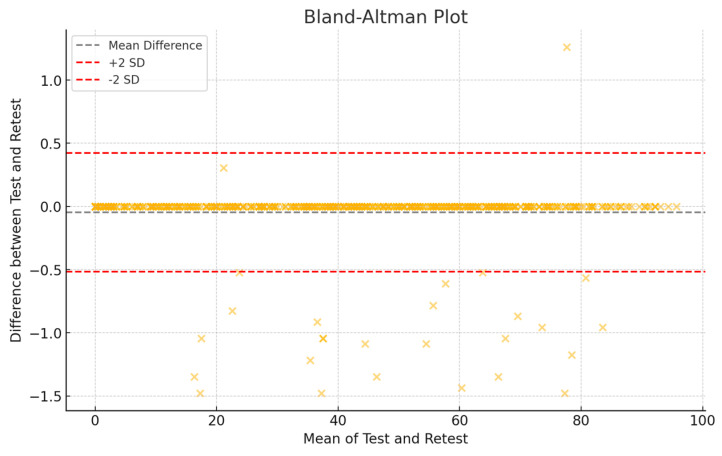
Bland–Altman plot for test–retest measurements.

The FFI-Sp-RA version showed good test–retest reliability, with a global ICC 2.1 of 0.89 (95% CI; 0.84–0.92). The SEM and MDC were 2.41 and 6.5, respectively (Figure 3).

The FFI-Sp-RA version demonstrated excellent internal consistency, with a Cronbach’s alpha result of 0.96.

### 3.2. Convergent Validity

The Pearson’s correlation coefficient between the FFI-Sp-RA and the VAS general and foot-specific pain was 0.608 (*p* < 0.001) and 0.719 (*p* < 0.001).

No ceiling effect (5.5%) or floor effect (3.4%) was observed in the FFI-Sp-RA total score. The 15% threshold was not exceeded in any case.

## 4. Discussion

The primary objective of our investigation was to evaluate the sensitivity of the Spanish version of the Foot Function Index for patients with rheumatoid arthritis (RA), aiming to offer a tool that captures the multifaceted impacts of RA on foot functionality.

Originally developed by Budiman-Mak et al. in 1991, the FFI was intended to measure the effects of RA on foot functionality through a 23-item questionnaire. The evolution of this tool, including its modifications to address prior deficiencies and the addition of new items, underscores the ongoing effort to refine patient-reported outcome measures (PROMs) for RA [9].

Subsequent modifications were made to the original framework to rectify identified shortcomings, culminating in the development of the Revised Foot Function Index (FFI-R). This revision involved the addition of new items and the differentiation of the instrument into two distinct versions: the FFI-R Long (FFI-RL), comprising 68 items, and the FFI-R Short (FFI-RS), encompassing 34 items [10].

The FFI has been compared for strengths and sensitivities with other PROMs. The findings of our study underscore the FFI’s comparative advantage in sensitivity over other PROMs like the Leeds Foot Impact Scale (LFIS) [20] and the Foot Health Status Questionnaire (FHSQ) for evaluating foot functionality and the progression of RA-related foot pathologies [21]. The FFI’s focus on pain, disability, and activity limitation offers a comprehensive framework for assessing the impact of RA, despite its noted lower sensitivity to change in comparison to the FHSQ.

The refinement of the FFI-Sp into a 15-item construct, through the exclusion of items that either confused participants or overlapped significantly with others, represents a critical step towards optimizing this tool for clinical and research purposes. Our analysis led to the exclusion of specific items from the pain and disability subscales, addressing issues related to clarity and redundancy.

The results have affirmed the retention of the FFI’s tripartite structure, encompassing subscales for pain (seven items), disability (six items), and activity limitation (two items), culminating in a revised 15-item FFI-Sp-AR version through the exclusion of eight items: FFI-5, FFI-6, FFI-10, FFI-12, FFI-15, FFI-19, FFI-20, and FFI-21. This exclusion was predicated on the elimination of items that induced confusion or redundancy, particularly those related to footwear and home ambulation, which did not contribute additional analytical clarity or value. Furthermore, items such as FFI-19, FFI-20, and FFI-21 were removed due to their exclusive association with RA flare-ups, failing to directly reflect foot functionality. This refinement and adaptation to the Spanish context, mirroring the approach taken in the Italian adaptation, underscore the necessity of a clear, concise, and culturally attuned instrument for assessing foot functionality in RA patients. It aligns the tool more closely with the specific experiences and needs of the patient population, enhancing its relevance and applicability in clinical and research settings [22].

The excellent internal consistency and convergent validity of our study align with those of other transcultural adaptations of the FFI, supporting the reliability and applicability of the FFI-Sp-RA version across different contexts. The large sample size of our study significantly enhances the robustness of our findings, reducing the potential for error and providing a solid foundation for the FFI’s utility in assessing the impact of RA on foot functionality.

In sociocultural adaptations such as the Spanish [12] and Turkish [23] versions for patients with musculoskeletal disorders in the foot and ankle, the French version for patients with rheumatoid arthritis [24], and the Norwegian version for patients with fasciopathy [25], among others, the 23 items are retained, showing good correlation coefficients but lower internal consistency compared to our results.

Our study confirms that the FFI-Sp-RA version demonstrates excellent test–retest reliability, greater internal consistency, and improved construct validity, similar to the sociocultural adaptation and modification to German (FFI-D18) performed with foot surgery patients [26] and the Italian version (FFI-I17) with patients having musculoskeletal disorders of the foot and ankle [22].

Items removed from the pain subscale include FFI-5 and FFI-6, similar to the Italian version FFI-I17 [22], which also removed FFI-7 and FFI-8, as in the German version [26].

From the disability subscale, items FFI-10, FFI-12, and FFI-15 were eliminated. The latter two, when reviewing the Spanish version [12], showed higher values in the pain subscale (FFI-12: 0.695, FFI-15: 0.703) than in the disability subscale (FFI-12: 0.560, FFI-15: 0.482), distorting the results at this sublevel.

In the activity limitation subscale, items FFI-19, FFI-20, and FFI-21 were removed. The Italian version [22] eliminated FFI-19 and FFI-20, while the German version [26] removed the entire subscale.

Additionally, the adaptation of the FFI for a Spanish-speaking RA population, while successful, highlights the need for ongoing validation and refinement of the tool across different languages and cultural contexts. This endeavor will ensure the FFI remains a valid and reliable measure for assessing the impact of RA on foot functionality globally.

The adapted version of the FFI for patients with rheumatoid arthritis allows for a more precise assessment of foot functionality, facilitating personalized intervention planning and progress monitoring, thereby improving patients’ quality of life and guiding clinical decision-making. The results can be generalized to other populations with similar conditions, though additional studies are needed to confirm its validity and explore its applicability in different clinical contexts.

This study’s results will enable more accurate diagnoses, tailored treatments, and improved patient monitoring for foot and ankle disorders, ensuring reliable assessments across diverse populations and enhancing overall clinical outcomes.

While our study provides valuable insights, it is not without limitations. The notable gender disparity among participants reflects the higher prevalence of RA in women, which could introduce bias in the findings. Future research should aim to address this limitation by seeking more balanced gender representation among participants.

## 5. Conclusions

In conclusion, our study not only reaffirms the value of the FFI as a valid and reliable tool for evaluating the impact of RA on foot functionality but also contributes to its ongoing refinement and adaptation for broader applicability. The modifications made to the FFI based on our findings enhance its clarity and relevance, making it an even more effective instrument for both clinical assessment and research into RA-related foot pathologies.

## Figures and Tables

**Table 1:** Characteristics of participants.

	Mean	IC 95%		SD
Age (years)	59.90	58.56	60.64	12.37
Weight (kg)	71.46	70.15	72.77	15.64
Height (cm)	162.32	161.45	163.18	10.30
Evolution (years)	15.93	15.00	16.85	11.05
FFI TOTAL	41.21	39.06	43.36	25.63
FFI Pain	43.34	41.25	45.43	24.96
FFI Disability	40.76	38.36	43.17	28.66
FFI Physical_Activity	10.69	9.63	11.75	12.60

SD—standard deviation. IC—interval confident.

**Table 2:** Factor matrix for the 23-item FFI Sp questionnaire.

	Factor 1	Factor 2	Factor 3
FFI_1 Intensity of maximum foot pain	0.808		
FFI_2 Does your foot hurt in the morning?	0.775		
FFI_3 Foot pain when walking?	0.809		
FFI_4 Pain when standing?	0.808		
FFI_7 Pain when walking with insoles?	0.844		
FFI_8 Pain when standing with insoles?	0.843		
FFI_9 Level of pain at the end of the day?	0.769		
FFI_11 Do you have difficulty walking on the street?		0.780	
FFI_13 Do you have difficulty climbing stairs?		0.824	
FFI_14 Do you have difficulty descending stairs?		0.816	
FFI_16 Do you have difficulty getting up from a chair?		0.804	
FFI_17 Do you have difficulty stepping up onto a curb?		0.820	
FFI_18 Do you have difficulty walking quickly?		0.793	
FFI_22 Did you use an assistive device inside the house?			0.916
FFI_23 Did you use an assistive device outside the house?			0.919

**Table 3:** Confirmatory factor analysis (factor loadings).

Factor	Indicator	Estimate	SE	Z	*p*
Factor 1	FFI_1	2.98	0.112	26.6	<0.001
	FFI_2	2.90	0.121	24.1	<0.001
	FFI_3	3.08	0.112	27.4	<0.001
	FFI_4	3.06	0.119	25.8	<0.001
	FFI_7	3.35	0.177	18.9	<0.001
	FFI_8	3.39	0.182	18.6	<0.001
	FFI_9	3.09	0.114	27.2	<0.001
Factor 2	FFI_11	3.11	0.124	25.1	<0.001
	FFI_13	3.41	0.122	28.0	<0.001
	FFI_14	3.37	0.124	27.1	<0.001
	FFI_16	2.88	0.121	23.7	<0.001
	FFI_17	3.05	0.121	25.2	<0.001
	FFI_18	3.46	0.136	25.5	<0.001
Factor 3	FFI_22	2.68	0.116	23.1	<0.001
	FFI_23	3.26	0.130	25.2	<0.001

## Data Availability

The data presented in this study are available on request from the corresponding author due to it being part of an unpublished thesis currently under embargo.

## Supplementary Materials

The following supporting information can be downloaded at: https://www.mdpi.com/article/10.3390/medicina60081339/s1, File S1: Foot Function Index versión española FFI-Sp.
