# Supplementary material for: Update of Modified Version of the Foot Function Index Tool Spanish Version (FFI-Sp), in Patients with Rheumatoid Arthritis: Cross Sectional Study
Source: Medicina (Kaunas). 2024 Aug 18;60(8):1339. doi: 10.3390/medicina60081339 (PMC11356240; doi:10.3390/medicina60081339)
Supplement: Supplementary file 1 [file medicina-60-01339-s001.zip › medicina-3097167-supplementary.pdf]

**Foot Function Index versión española FFI-Sp**												
Número de días con dolor de pie (ponga 0 si no ha tenido dolor reciente): ________________												
Por favor conteste todas las preguntas. Puntúe en función de su pie **durante la SEMANA pasada**, según las instrucciones en cada uno de los apartados. Por favor lea cada pregunta y marque un número del 0 al 10.												
**Escala del dolor.**												
**Sin Dolor 0 1 2 3 4 5 6 7 8 9 10 Máximo dolor imaginable**												
1	¿Intensidad del máximo dolor del pie?	0	1	2	3	4	5	6	7	8	9	10
2	¿Le duele el pie por la mañana?	0	1	2	3	4	5	6	7	8	9	10
3	¿Dolor del pie al caminar?	0	1	2	3	4	5	6	7	8	9	10
4	¿Dolor al estar de pie?	0	1	2	3	4	5	6	7	8	9	10
5	¿Dolor al caminar con zapatos?	0	1	2	3	4	5	6	7	8	9	10
6	¿Dolor al permanecer de pie con zapatos?	0	1	2	3	4	5	6	7	8	9	10
7	¿Dolor al caminar con plantillas?	0	1	2	3	4	5	6	7	8	9	10
8	¿Dolor al permanecer de pie con plantillas?	0	1	2	3	4	5	6	7	8	9	10
9	¿Nivel de dolor al final del día?	0	1	2	3	4	5	6	7	8	9	10
**Escala de discapacidad.**												
**Sin dificultad 0 1 2 3 4 5 6 7 8 9 10 Dificultad extrema que imposibilita la función**												
10	¿Tiene dificultad al andar en casa?	0	1	2	3	4	5	6	7	8	9	10
11	¿Tiene dificultad al andar por la calle?	0	1	2	3	4	5	6	7	8	9	10
12	¿Tiene dificultad al andar 500 metros?	0	1	2	3	4	5	6	7	8	9	10
13	¿Tiene dificultad al subir escaleras?	0	1	2	3	4	5	6	7	8	9	10
14	¿Tiene dificultad al bajar escaleras?	0	1	2	3	4	5	6	7	8	9	10
15	¿Tiene dificultad al estar de puntillas?	0	1	2	3	4	5	6	7	8	9	10
16	¿Tiene dificultad al levantarse de la silla?	0	1	2	3	4	5	6	7	8	9	10
17	¿Tiene dificultad al subir el bordillo de la acera?	0	1	2	3	4	5	6	7	8	9	10
18	¿Tiene dificultad al andar rápido?	0	1	2	3	4	5	6	7	8	9	10
**Escala de limitación de la actividad.**												
**Nunca 0 1 2 3 4 5 6 7 8 9 10 Siempre**												
19	¿Permaneció e casa todo el día debido a los pies?	0	1	2	3	4	5	6	7	8	9	10
20	¿Permaneció en la cama todo el día debido a los pies?	0	1	2	3	4	5	6	7	8	9	10
21	¿Limitó sus actividades debido a sus pies?	0	1	2	3	4	5	6	7	8	9	10
22	¿Hizo uso de un dispositivo de ayuda (bastón, andador, muleta, etc) dentro de su casa?	0	1	2	3	4	5	6	7	8	9	10
23	¿Hizo uso de un dispositivo de ayuda (bastón, andador, muleta, etc) dentro de su casa?	0	1	2	3	4	5	6	7	8	9	10

Resultado: (_______/230) x 100:_____%

Foot Function Index versión española modificada FFI-Sp-RA												
Número de días con dolor de pie (ponga 0 si no ha tenido dolor reciente):________________												
Por favor conteste todas las preguntas. Puntúe en función de su pie **durante la SEMANA pasada**, según las instrucciones en cada uno de los apartados. Por favor lea cada pregunta y marque un número del 0 al 10.												
**Escala del dolor.**												
**Sin Dolor 0 1 2 3 4 5 6 7 8 9 10 Máximo dolor imaginable**												
1	¿Intensidad del máximo dolor del pie?	0	1	2	3	4	5	6	7	8	9	10
2	¿Le duele el pie por la mañana?	0	1	2	3	4	5	6	7	8	9	10
3	¿Dolor del pie al caminar?	0	1	2	3	4	5	6	7	8	9	10
4	¿Dolor al estar de pie?	0	1	2	3	4	5	6	7	8	9	10
5	¿Dolor al caminar con plantillas?	0	1	2	3	4	5	6	7	8	9	10
6	¿Dolor al permanecer de pie con plantillas?	0	1	2	3	4	5	6	7	8	9	10
7	¿Nivel de dolor al final del día?	0	1	2	3	4	5	6	7	8	9	10
**Escala de discapacidad.**												
**Sin dificultad 0 1 2 3 4 5 6 7 8 9 10 Dificultad extrema que imposibilita la función**												
8	¿Tiene dificultad al andar por la calle?	0	1	2	3	4	5	6	7	8	9	10
9	¿Tiene dificultad al subir escaleras?	0	1	2	3	4	5	6	7	8	9	10
10	¿Tiene dificultad al bajar escaleras?	0	1	2	3	4	5	6	7	8	9	10
11	¿Tiene dificultad al levantarse de la silla?	0	1	2	3	4	5	6	7	8	9	10
12	¿Tiene dificultad al subir el bordillo de la acera?	0	1	2	3	4	5	6	7	8	9	10
13	¿Tiene dificultad al andar rápido?	0	1	2	3	4	5	6	7	8	9	10
**Escala de limitación de la actividad.**												
**Nunca 0 1 2 3 4 5 6 7 8 9 10 Siempre**												
14	¿Hizo uso de un dispositivo de ayuda (bastón, andador, muleta, etc) dentro de su casa?	0	1	2	3	4	5	6	7	8	9	10
15	¿Hizo uso de un dispositivo de ayuda (bastón, andador, muleta, etc) dentro de su casa?	0	1	2	3	4	5	6	7	8	9	10

Resultado: (________/150) x 100: ______%
